# Unusual presentation of extra-nodal double-hit follicular lymphoma: a case report

**DOI:** 10.1186/s12876-022-02308-8

**Published:** 2022-05-20

**Authors:** Stefano Lazzi, Massimo Granai, Marco Capanni, Falko Fend

**Affiliations:** 1 Department of Medical Biotechnology, University of Siena, Siena, Italy; 2 Institute of Pathology and Neuropathology, Tübingen University Hospital and Comprehensive Cancer Center Tübingen-Stuttgart, Tübingen, Italy; 3 General Surgery Unit, USL 7, Siena, Italy

**Keywords:** Colonoscopy, Gastrointestinal lymphoma, Follicular lymphoma, Double-hit follicular lymphoma, Case report

## Abstract

**Background:**

To the best of our knowledge, this case represents the first report of an extranodal double-hit follicular lymphoma (DH-FL) as an intestinal polypoid lesion.

**Case presentation:**

A 72-year-old woman presents with constipation. Colonoscopy reveals a sessile polypoid lesion of the colon bearing morphological, immunohistochemical and molecular hallmarks of DH-FL. Complete clinical staging and bone marrow biopsy showed no signs of disseminated disease. The patient, after two years of follow-up is still free of disease confirming the indolent behaviour of this limited lesion.

**Conclusions:**

A synoptic view at all the features of the patient and not merely at the molecular hallmarks of a disease are essential to establish the correct clinical approach.

## Background

Primary double-hit follicular lymphoma (DH-FL) is rare. We describe the first case of follicular lymphoma with double-hit cytogenetics as a primary colonic localization. Moreover, our study discusses the clinical impact of MYC translocation in FL given the fact that data reported in literature are scarce. So far, DH-FL does not belong to high-grade B-cell lymphoma with MYC and BCL2 and/or BCL6 rearrangement category according to the revised 2017 WHO classification and no consensus exists on how to manage patients with this disease. Our case prompts the necessity of a re-classification of such entities.

## Case presentation

**Fig. 1:**
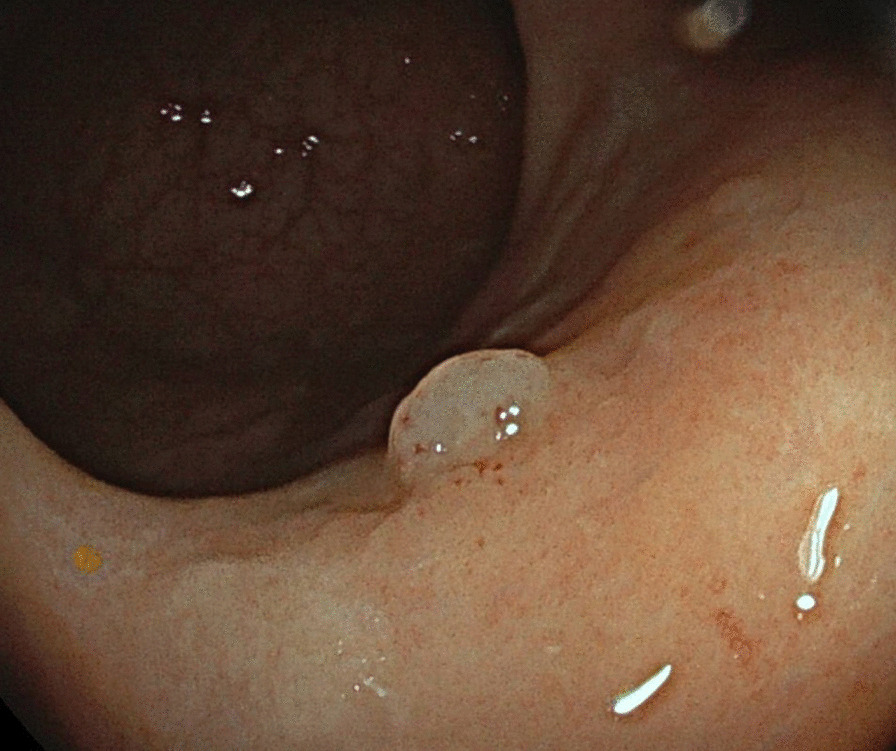
A sessile polypoid lesion of 5 mm in sigmoid colon was found by colonoscopy. The polyp had a smooth surface, without nodularity or ulceration

**Fig. 2:**
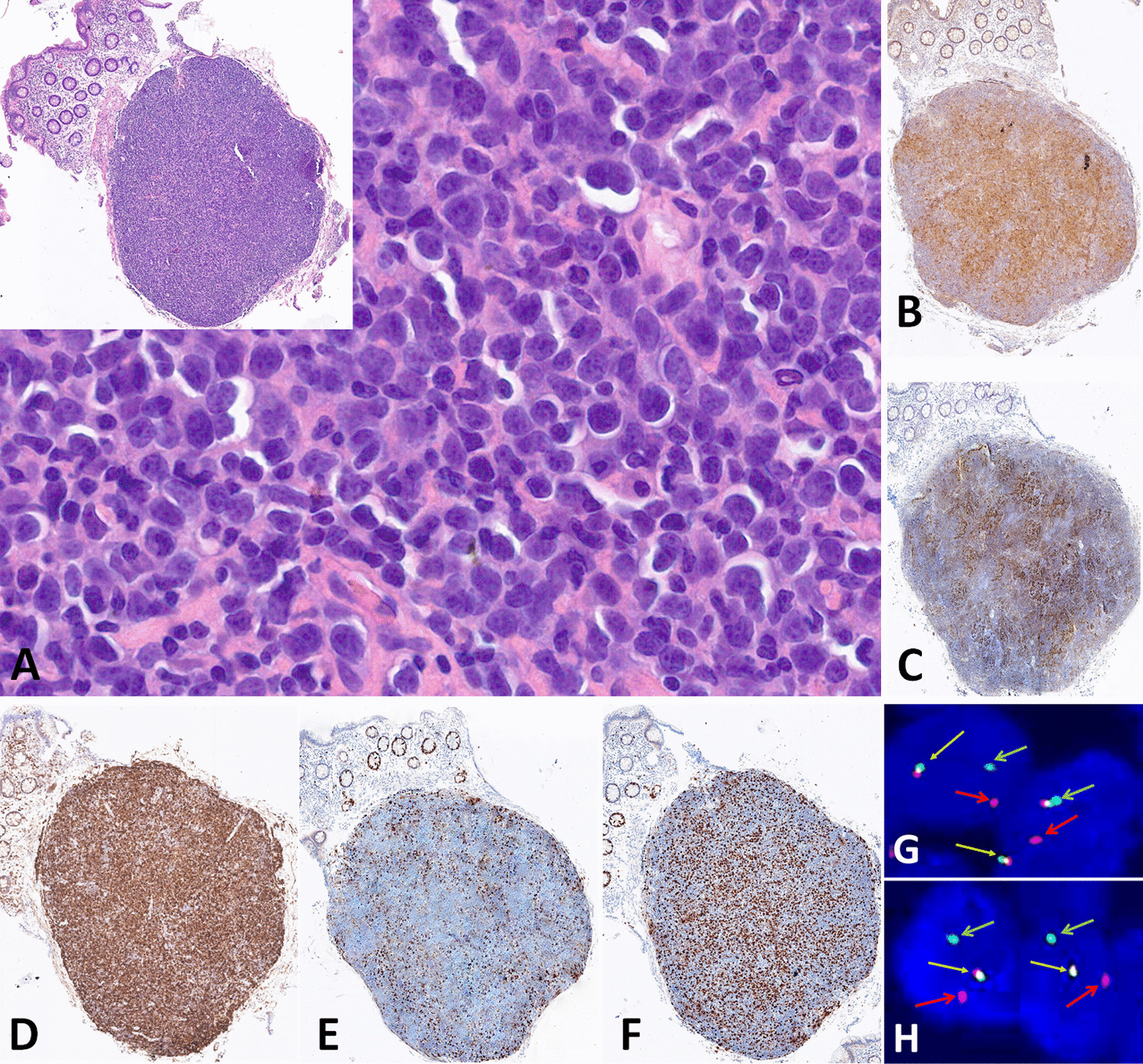
Lymphoid infiltrate with follicular architecture (**A** inset), characterized by large centroblasts with few, centrocytes (**A**). The lymphoid cells were positive for CD10 (**B**) with remnants of the FDC meshwork highlighted by CD21 (**C**) staining. BCL6 (**D**), BCL2 (**E**) were also positive. The atypical cells exhibited MYC protein (**F**). Fluorescent in-situ hybridization (FISH) studies detected both *IGH/BCL2* (**G**) and *IGH/MYC* (**H**) gene rearrangements in approximately 60% of interphase nuclei with dual-colour break-apart probes (**G**–**H**, Yellow arrows: normal alleles; red and green arrows: breakages). [original magnification: **A** inset, **B**–**F**: × 40; **A**: × 400; **G**,**H**: × 1000]

A 72-year-old woman presented with complaints of constipation for three months. Physical examination, haematological and biochemical investigations were normal. Medical history was unremarkable. A colonoscopy was performed and revealed a sessile polypoid lesion of 5 mm of the sigmoid colon. The polyp had a smooth surface, without nodularity or ulceration (Fig. 1). Histopathological examination of the biopsy showed a submucosal lymphoid infiltrate with follicular architecture (Fig. 2A inset), characterized by large centroblasts with few, centrocytes (Fig. 2A). Immunohistochemistry showed that the lymphoid cells were positive for CD20, CD10, BCL6, BCL2, and MYC protein expression with remnants of the FDC meshwork highlighted by CD21 staining and high proliferation index (Ki-67: 60–70%) (not shown) (Fig. 2B–F). Fluorescent in-situ hybridization (FISH) studies (probes: Vysis LSI *MYC* Dual Color Break Apart Rearrangement Probe; Vysis LSI *BCL2* Dual Color Break Apart FISH Probe; Abbott, Chicago, Illinois) detected both IGH*/BCL2* (Fig. 2G) and IGH/*MYC* (Fig. 2H) gene rearrangements in approximately 60% of interphase nuclei (Fig. 2G–H, Yellow arrows: normal alleles; red and green arrows: breaks, original magnification × 1000). No evidence of *BCL6* gene rearrangement was found (probe: Vysis LSI *BCL6* Dual Color Break Apart Rearrangement Probe; Abbott). The diagnosis of an extra-nodal follicular lymphoma grade 3A with *BCL2* and *MYC* rearrangements was made (DH-FL). The patient underwent a complete clinical staging with FDG-PET/CT and bone marrow biopsy. No signs of disseminated disease were found and primary colonic localization was confirmed. Although bearing molecular hallmarks of malignancy, a watch-and-wait approach was adopted according to the clinically favourable setting. The patient, after two years of follow-up shows no signs of relapse.

## Discussion and conclusions

Primary FLs are mostly detected in the lymph nodes; although relatively rare, they can appear in the gastrointestinal tract, being the most common site for extranodal lymphoma. Intestinal FLs account for only 1.0 to 3.6% of cases, and the duodenum is the most common site of occurrence, occurring rarely in the colorectal area [1].

Rarely, follicular lymphoma may arise with MYC and BCL2 and/or BCL6 rearrangements, termed double-hit follicular lymphoma or triple-hit follicular lymphoma and should not be mistaken with the previously described double-hit lymphoma (DHL). While DHL classically presents with high-grade morphology resembling diffuse large B-cell lymphoma and/or Burkitt lymphoma with clinically aggressive course, DH-FL demonstrates the same translocation patterns but exhibits a follicular lymphoma histology, with no areas of high-grade cytologic transformation [2].

Primary DH-FL is rare. So far, very few cases have been described, however presenting with nodal localization [3, 4]. To the best of our knowledge, this is the first case of follicular lymphoma with double-hit cytogenetics with primary extranodal presentation.

For the diagnosis of such entities FISH is mandatory. However, such technique is not routinely used for localized lesions with a small cell lymphoma morphology. On the other hand, high grade morphology, high proliferation index and high MYC protein expression can prompt the pathologist for in-deep molecular analysis as in our case.

This diagnosis offers a clinical challenge, given the difficulty of predicting whether the tumor will behave aggressively, as suggested by its cytogenetic profile or indolently, as indicated by its limited stage and preserved follicular growth pattern.

Some authors have shown that *MYC* and *BCL2* double-hit cytogenetic profile in FL favours the transformation to high-grade lymphoma and a poor outcome [5]. Conversely, other studies, demonstrated that DH-FL patients show a tendency to reach complete or partial remission with longer survival [6, 7]. However, it is difficult to draw definitive conclusions regarding survival of DH-FL patients, as data reported in literature are controversial and limited to small studies. According to the revised 2017 WHO classification of lymphoid neoplasms, the double hit category includes only high-grade B-cell lymphoma with diffuse growth pattern, currently excluding DH-FL [2]. Whether DH-FL should be included in this category remains controversial, given the limited knowledge about the potential clinical impact of *MYC* translocation in FL [5]. The patient, after two years of follow-up is still healthy and free of disease confirming the indolent behaviour of this limited lesion.

This case illustrates that a synoptic view at all the features of the disorder and not at isolated markers are essential to establish a tailored clinical approach and to avoid overtreating.

## Data Availability

Not applicable.

## Abbreviations

DH-FL: Double-hit follicular lymphoma.
FDG-PET: Fluorodeoxyglucose (FDG)-positron emission tomography.
CT: Computer tomography
